# Relationship Between Atrial Fibrillation Recurrence and Frontal QRS-T Angle After Effective Cardioversion

**DOI:** 10.7759/cureus.33541

**Published:** 2023-01-09

**Authors:** Kaya Özen, Mehmet Zülkif Karahan

**Affiliations:** 1 Cardiology, Gazi Yaşargil Training and Research Hospital, Diyarbakır, TUR; 2 Cardiology, Mardin Artuklu University Faculty of Medicine, Mardin, TUR

**Keywords:** ecg parameter, frontal qrs-t angle, recurrence, atrial fibrillation, cardioversion

## Abstract

Objective: Maintaining sinus rhythm is important in the management of atrial fibrillation (AF). After cardioversion, there is a significant probability of AF recurrence. There is limited research on the relationship between AF recurrence and ECG parameters. This study aimed to evaluate whether the frontal plane QRS-T angle (fQRS-T), a predictor of ventricular heterogeneity, could be used to predict AF recurrence following cardioversion.

Methods: The study was conducted as a retrospective observational study. Patients diagnosed with acute-onset AF for the first time were included in the study. All patients underwent an ECG after cardioversion, and ECG parameters were evaluated. The patients were separated into two groups based on the presence of AF recurrence during hospitalization after cardioversion. The relationship between the fQRS-T and AF recurrence was also examined.

Results: A total of 162 patients, comprising 68 women (41.9%) and 94 men (58.1%) with an average age of 59.4±6.5 years, were enrolled in the research. Based on the patient monitoring device findings, patients were separated into two groups: non-recurrent AF (n=118) and recurrent AF (n=44). P-wave duration was significantly longer in the recurrence group (p=0.009). The recurrence group's mean fQRS-T was significantly higher (p<0.001). AF recurrence was substantially higher in patients with fQRS-T >90 ° compared to those with fQRS-T ≤90 ° (56.1% vs. 14.2%, p <0.001). Increased fQRS-T>93.7 ° indicated AF recurrence with 78.3% sensitivity and 83.4% specificity (AUC {area under curve}:0.748, p < 0.001). In multivariate analysis, fQRS-T was revealed to be an early indicator of recurrent AF (OR: 1.882, 95%CI: 1.358-2.881, p<0.001).

Conclusion: The fQRS-T, an easily determinable parameter from automatic identification ECG recordings, may be useful for predicting the early return of AF after successful cardioversion.

## Introduction

Atrial fibrillation (AF) is a frequent atrial arrhythmia characterized by disorganized and fast atrial excitation, leading to the lack of mechanical atrial systole and irregular ventricular activation [1]. The resulting irregularity causes cardiac adverse events. Ischemic stroke, cardiac failure, and death are unavoidable after the onset of atrial fibrillation [2,3]. Conversion of atrial fibrillation to sinus rhythm with cardioversion is a cornerstone of the management of acute-onset AF [4]. Unfortunately, AF recurrence is relatively prevalent in clinical practice, and maintaining sinus rhythm is difficult.

P-wave duration from electrocardiography (ECG) parameters was used to predict the development of AF and the risk of AF recurrence [5]. P-wave peak time (PWPT) is an ECG measurement that represents the period between the conduction of electrical activity from the sinoatrial node and the greatest positive summation from both atriums [6]. P-wave terminal force in lead V1 (PTFV1) is a classic ECG diagnostic for abnormalities of the left atrium [7]. In previous studies, these parameters have been shown to be directly related to the risk of AF.

The frontal plane QRS-T angle (fQRS-T), which is the differential between the QRS and T axes, is a novel marker indicating heterogeneity in ventricular repolarization and is auto-detected on an ECG [8]. Increased fQRS-T has been associated with adverse events [9]. However, few studies have examined the correlation between the fQRS-T and AF. In this study, we aimed to research whether the fQRS-T predicts early AF recurrence following effective cardioversion.

## Materials and methods

Study design and subject

This research was carried out retrospectively at Gazi Yasargil Training and Research Hospital, a tertiary center. Patients admitted to this study were >18 years of age and were admitted with acute-onset AF between 2020 and 2022. AF was defined according to the 2021 ESC (European Society of Cardiology) guideline [10]. Patients were included within 24 hours of AF onset. In total, 178 consecutive patients with AF were included in this study. Patients with preexisting AF, pacemaker rhythm, bundle branch block, hyperthyroidism, renal failure, active cancer, pulmonary disease, and congenital heart disease were excluded from the study. Excluded from the research were patients whose data were unavailable and whose ECG analysis failed. The protocol was accepted by the ethics committee at Gazi Yaşargil Training and Research Hospital (No: 2022-222, Date: 25/11/2022). It conforms to the Declaration of Helsinki's ethical criteria 2013.

Study protocol

**Figure 1:**
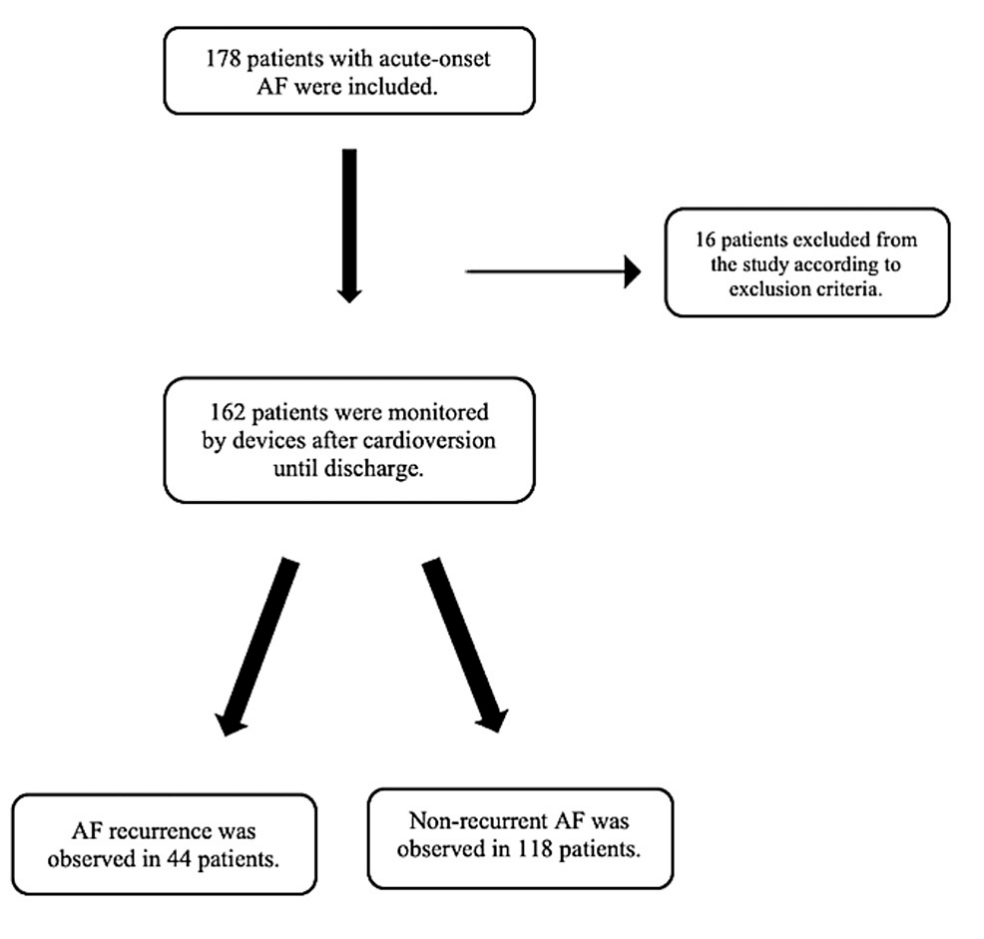
Flow diagram of the study

All patients included in the study had a blood test and an electrocardiograph (model ECG-1350K Nihon-Kohden Corporation) at 25 mm/s and 10 mm/mV amplitude after cardioversion. According to hospital archive records, all patients were followed up in the hospital after cardioversion until discharge. Depending on monitoring findings, the individuals were classified into two groups: those with non-recurrent AF and those with recurrent AF. Figure 1 shows a flow diagram of the patients.

Definitions

**Figure 2:**
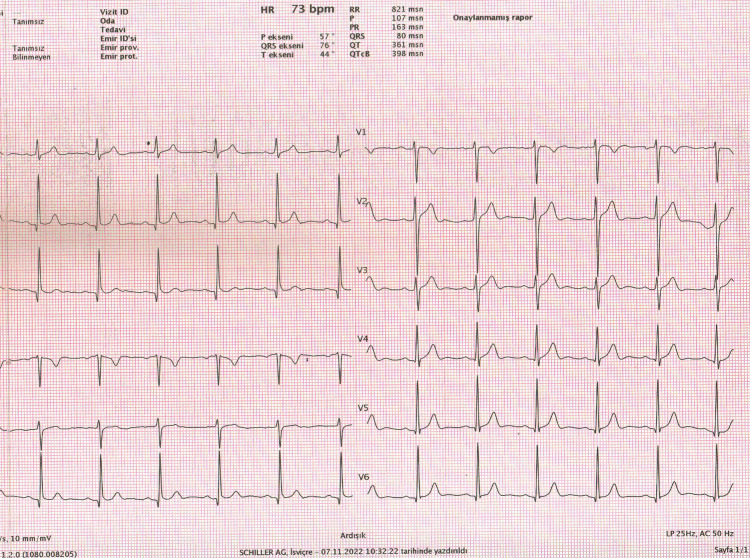
Calculation of frontal QRS-T angle from automated surface ECG report Frontal QRS-T angle (fQRS-T) = QRS axis - T axis = 76° - 44° = 32°

The ECG was performed by qualified and knowledgeable staff. Two experienced observers who were blinded to participant information assessed ECGs. The fQRS-T was defined as the difference between the QRS and T axes (Figure 2).

The lowest angle (360°) was utilized when the angle was greater than 180 degrees (°). The fQRS-T was formed using ECG data acquired automatically. Clinically, AF is an asymptomatic or symptomatic that is diagnosed by surface ECG. At least 30 seconds or a complete 12-lead ECG is necessary to diagnose clinical AF. Subclinical AF is characterized by AF episodes detected by patient monitoring devices in individuals without AF symptoms and in whom clinical AF has not been diagnosed before. In our study, recurrent AF was defined as a new atrial fibrillation event occurring in the hospital after the incident event. Cardioversion includes both pharmacological drug administration and electrical shock therapy.

Statistics

The IBM SPSS 24.0 package software (IBM Corp., Armonk, NY) was applied for the analysis. The initial continuous variables are presented as mean ± standard deviation or median (interquartile range). The normality distribution of the variables was analyzed utilizing the Kolmogorov-Smirnov, and Shapiro-Wilk tests. Frequencies and percentages were used to represent categorical variables. The Chi-square test or Fisher’s exact test was utilized for categorical variables. Student’s t-test or Mann-Whitney U-test was utilized to compare continuous variables, as appropriate. Multivariate logistic regression analysis was used to assess the independent predictors of covariates that cause recurrent AF. In the multivariate model, covariates with p-values <0.05 were included. If the p-value was less than 0.05, then all tests were deemed statistically significant.

## Results

A total of 162 patients, comprising 68 women (41.9%) and 94 men (58.1%) with an average age of 59.4±6.5 years, were enrolled in the research. Based on the patient monitoring device findings, patients were separated into two groups non-recurrent AF (n=118) and recurrent AF (n=44). The patients' baseline demographic and hematological parameters are shown in Table 1 and Table 2 respectively.

**Table 1:** Clinical characteristics of the patients Data are expressed as mean SD, number (percentage), or median (interquartile range) as appropriate. AF: atrial fibrillation; DL: dyslipidemia; LVEF: left ventricular ejection fraction; LAD: left atrial diameter; QTc: corrected-QT; fQRS-T: frontal plane QRS-T angle

PARAMETERS	Recurrent AF N=44	Non-recurrent AF N=118	P-Value
Age (Years)	63.1±6.2	58.2±7.6	<0.001
Gender, female, n (%)	19 (43.1)	49 (41.5)	0.612
Hypertension, n (%)	17 (38.6)	45 (38.1)	0.866
Diabetes mellitus, n (%)	15 (34)	36 (30.5)	0.328
DL, n (%)	10 (22.7)	27 (22.8)	0.910
Smoking, n (%)	25 (56.8)	54 (45.7)	0.121
Hospital stay (hour)	37.2±5.6	34.6±6.4	0.159
LVEF (%)	52.1±5.6	55.3±6.2	0.568
LAD (mm)	39.2±3.8	40.2±3.6	0.783
Heart rate (beats/min.)	82.3±7.8	81.2±7.2	0.487
P wave duration (ms)	112±9.7	106±11.2	0.009
QRS duration (ms)	93.5±5.2	92.1±4.5	0.239
QT interval (ms)	381±20	378±21	0.205
QTc interval (ms)	442±7.6	440±11.2	0.286
fQRS-T (degree)	90±35.6	51±38.4	<0.001
Medications;			
Beta-blockers, n (%)	28 (63.6)	78 (66.1)	0.506
Calcium channel blockers, n (%)	16 (36.3)	40 (33.9)	0.714

The patients with AF recurrence were older. In the recurrent group, the duration of the P-wave was considerably longer (p = 0.009). The recurrent AF group had a substantially higher mean fQRS-T (p<0.001). There were no substantial differences in hematological and biochemical parameters as shown in Table 2.

**Table 2:** Hematological and biochemical parameters of patients Data are expressed as mean ± SD and median (interquartile range) as appropriate. AF: atrial fibrillation; WBC: white blood cell; AST: aspartate aminotransferase; ALT: aspartate aminotransferase; LDL: low-density lipoprotein; HDL: high-density lipoprotein; TSH: thyroid-stimulating hormone; eGFR: estimated glomerular filtration rate

PARAMETERS	Recurrent AF N:44	Non-recurrent AF N=118	P-Value
WBC count (×103/µL)	11.9±2.74	11.5±3.22	0.410
Hemoglobin (g/dl)	13.7±1.38	13.3±1.76	0.119
Platelet count (×103/µL)	256±58.5	253±67.2	0.845
Sodium (meq/L)	140 (5)	140 (3.75)	0.619
Potassium (meq/L)	4.23±0.44	4.11±0.63	0.224
Glucose (mg/dl)	122 (33.2)	118 (31.5)	0.333
Creatine (mg/dl)	0.86 (0.33)	0.88 (0.33)	0.801
AST (U/L)	21.7±5.19	22.1±5.92	0.648
ALT (U/L)	28.3±3.13	29.1±4.31	0.703
Triglycerides (mg/dl)	118 (63.2)	100 (44.5)	0.340
LDL (mg/dl)	112 (44.4)	105 (46.5)	0.154
HDL (mg/dl)	42 (17.3)	42 (14.3)	0.664
TSH (μIU/ml)	2.78±0.68	2.56±0.79	0.072
eGFR	96.5 (36.4)	94 (38.5)	0.925

AF recurrence was substantially higher in patients with fQRS-T >90° than in patients with fQRS-T ≤90° (56.1% vs. 14.2%, p <0.001) (Table 3).

**Table 3:** Clinical features of patients based on the fQRS-T Data are expressed as mean SD, number (percentage), or median (interquartile range) as appropriate. DL: dyslipidemia; LVEF: left ventricular ejection fraction; LAD: left atrial diameter; QTc: corrected-QT; fQRS-T: frontal plane QRS-T angle; AF: atrial fibrillation

PARAMETERS	fQRS-T ≤90 ° N=112	fQRS-T>90^0^ N=50	P-Value
Age (Years)	53.1±4.2	63.2±5.6	<0.001
Gender, female, n (%)	60 (53.6)	30 (60.5)	0.412
Hypertension, n (%)	43 (38.3)	19 (38.1)	0.916
Diabetes mellitus, n (%)	47 (41.8)	29 (58.1)	0.032
DL, n (%)	25 (28.1)	12 (24.2)	0.043
Smoking, n (%)	51 (45.5)	28 (56.1)	0.062
LVEF (%)	53.1±5.3	54.3±6.4	0.643
LAD (mm)	39.6±3.4	40.1±3.6	0.581
Heart rate (beats/min.)	81.3±8.8	83.2±9.2	0.287
P wave duration (ms)	102±8.7	123±7.2	<0.001
QRS duration (ms)	92.5±4.5	92.1±7.5	0.739
QT interval (ms)	381±22.7	374±11.1	0.061
QTc interval (ms)	442±9.6	439±9.9	0.183
fQRS-T (degree)	39±18.6	116±18.4	<0.001
AF recurrence, n (%)	16 (14.2)	28 (56.1)	<0.001

**Figure 3:**
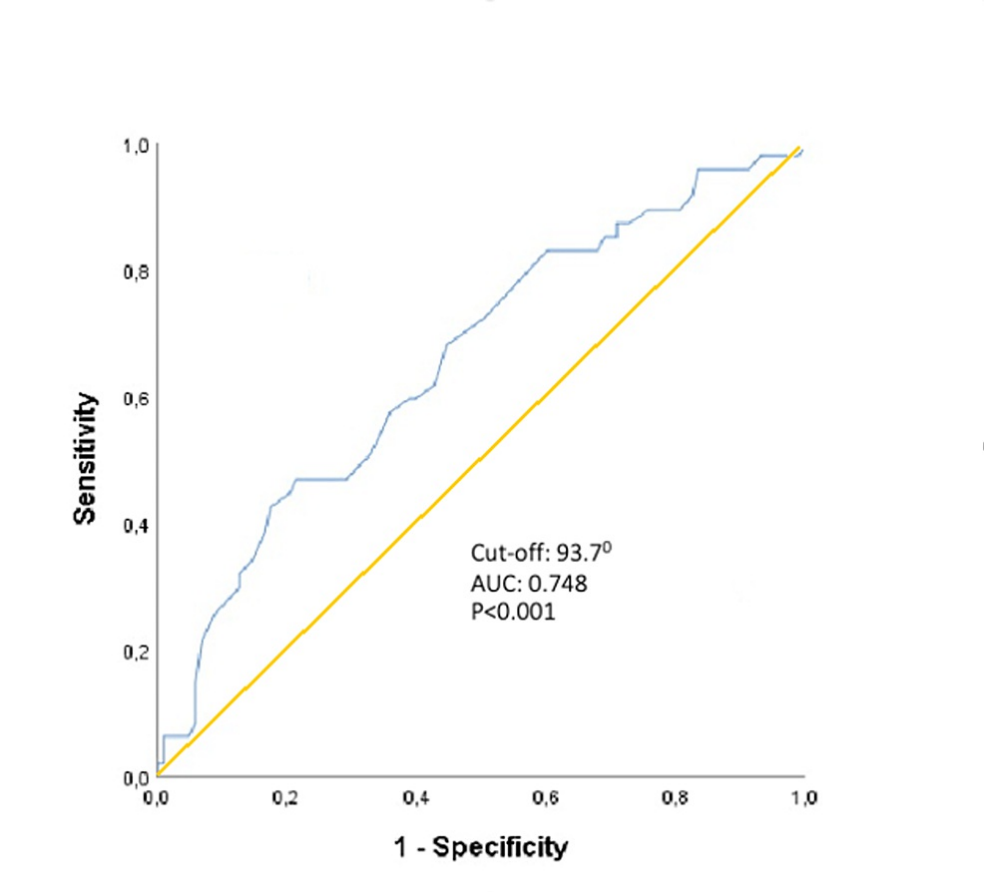
ROC curve of frontal QRS-T angle for the diagnosis of recurrent AF ROC, receiver operating characteristic; AF, Atrial fibrillation

Analysis of the receiver operating characteristic (ROC) curve revealed that an elevated fQRS-T >93.7° predicted AF recurrence with 78.3% sensitivity and 83.4% specificity (AUC: 0.748, p <0.001) (Figure 3).

Multivariate analysis was performed to determine independent predictors of recurrent AF. In multivariate analysis, fQRS-T (odds ratio {OR} {95%CI}, 1.882 {1.358-2.897}, p<0.001), age (OR {95%Cl}, 1.482 {1.172-2.182}, p<0.001) and p-wave duration (OR {95%Cl}, 1.192 {1.078-1.582}, p=0.032) were revealed to be independent predictors of recurrent AF (Table 4).

**Table 4:** Independent predictors for recurrent AF by multivariate analysis

Parameters	Multivariate analysis		
	OR	95% CI	P-Value
Age	1.482	1.172-2.182	<0.001
P wave duration	1.192	1.078-1.582	0.032
fQRS-T	1.882	1.358-2.897	<0.001

## Discussion

The results of the study demonstrated that the fQRS-T was an independent and powerful predictor of AF recurrence after cardioversion. The fQRS-T may be useful for monitoring acute-onset AF treatment in hospitalized patients.

AF is the most prevalent persistent atrial arrhythmia and is related to extremely high morbidity and mortality rates. AF has a complex and diverse etiology and is a significant cause of heart structure and function impairment [11,12]. Restoration and maintaining sinus rhythm as the most essential aspect of AF therapy are significantly associated with improved outcomes in selected patients. Pharmacological cardioversion is recommended more often than electrical cardioversion in treating acute-onset AF patients [13]. In our study, electrical cardioversion was not performed because the patients were hemodynamically stable.

Although ECG is an important factor in the follow-up of patients with AF, sufficient data on ECG parameters were not explained AF recurrence in patients with sinus restoration. The identification of new ECG parameters such as fQRS-T may be useful in predicting the development of acute AF in clinical practice. The fQRS-T, which is an indicator of heterogeneity in ventricular activation, defines the angular difference between ventricular depolarization and repolarization [14]. The QRS-T angle could be calculated using different measures. These are termed the spatial QRS-T and the frontal QRS-T. The measurement of the spatial QRS-T angle is quite complicated and requires sophisticated computer algorithms [15]. On the other hand, fQRS-T can be readily measurable from the automatic report section of the ECG equipment and well matches the spatial QRS-T angle for risk calculation. Despite the upper limit ranges for fQRS-T, the usual value is between 45° and 60° [16]. Numerous observational studies over the last decade have linked the QRS-T angle to cardiac mortality and other fatal consequences [17,18]. A larger fQRS-T angle suggests improper control of ventricular repolarization and is acknowledged as a significant and independent risk factor for cardiac arrhythmias in comparison to electrocardiographic risk indicators such as p-wave duration [19]. An angle >90° is associated with arrhythmic events and mortality [20].

In a study, the fQRS-T was shown to be a marker for newly diagnosed hypertensive patients [21]. In Zehir et al. study, individuals with slow coronary flow (SCF) and fQRS-T of more than 93° were more likely to have cardiac arrhythmia events [22]. Usalp et al. emphasized that the fQRS-T was higher in hypothyroidism patients who experienced dysrhythmia [23]. In another study, the fQRS-T predictive value in patients with NSTMI (non-ST-elevation myocardial infarction) and atrial fibrillation was 81° [24]. On the other hand, fQRS-T was not linked with dysrhythmia in patients with brucellosis, and vertigo in Günlü et al. studies [25,26].

Limitations

The population under research was small. The Holter rhythm was not conducted. The spatial QRS-T angle could not be calculated because there was no necessary computer program.

## Conclusions

Our study showed that increased fQRS-T, a marker that can be readily determined from automated ECG records, may be beneficial for predicting the early recurrence of AF following successful cardioversion in hospitalized patients. Further multi-center investigations are required for clinical application.
